# Hypothermia is not therapeutic in a neonatal piglet model of inflammation-sensitized hypoxia–ischemia

**DOI:** 10.1038/s41390-021-01584-6

**Published:** 2021-05-28

**Authors:** Kathryn A. Martinello, Christopher Meehan, Adnan Avdic-Belltheus, Ingran Lingam, Tatenda Mutshiya, Qin Yang, Mustafa Ali Akin, David Price, Magdalena Sokolska, Alan Bainbridge, Mariya Hristova, Ilias Tachtsidis, Cally J. Tann, Donald Peebles, Henrik Hagberg, Tim G. A. M. Wolfs, Nigel Klein, Boris W. Kramer, Bobbi Fleiss, Pierre Gressens, Xavier Golay, Nicola J. Robertson

**Affiliations:** 1 Institute for Women’s Health, University College London, London, UK; 2 Robinson Research Institute, University of Adelaide, Adelaide, SA Australia; 3 Department of Paediatrics, Ondokuz Mayıs University, Samsun, Turkey; 4 Medical Physics and Biomedical Engineering, University College London NHS Foundation Trust, London, UK; 5 Medical Physics and Biomedical Engineering, University College London, London, UK; 6 Adolescent, Reproductive and Child Health Centre, London School of Hygiene and Tropical Medicine, London, UK; 7 Department of Clinical Sciences, Centre of Perinatal Medicine and Health, Sahlgrenska Academy, Gothenburg University, Gothenburg, Sweden; 8 Centre for the Developing Brain, Kings College London, London, UK; 9 Department of Pediatrics, University of Maastricht, Maastricht, The Netherlands; 10 Paediatric Infectious Diseases and Immunology, Institute of Child Health, University College London, London, UK; 11 School of Health and Biomedical Sciences, RMIT University, Melbourne, VIC Australia; 12 Université de Paris, NeuroDiderot, Inserm, Paris, France; 13 Institute of Neurology, University College London, London, UK; 14 Centre for Clinical Brain Sciences, University of Edinburgh, Edinburgh, UK

## Abstract

**Background:**

Perinatal inflammation combined with hypoxia–ischemia (HI) exacerbates injury in the developing brain. Therapeutic hypothermia (HT) is standard care for neonatal encephalopathy; however, its benefit in inflammation-sensitized HI (IS-HI) is unknown.

**Methods:**

Twelve newborn piglets received a 2 µg/kg bolus and 1 µg/kg/h infusion over 52 h of *Escherichia coli* lipopolysaccharide (LPS). HI was induced 4 h after LPS bolus. After HI, piglets were randomized to HT (33.5 °C 1–25 h after HI, *n* = 6) or normothermia (NT, *n* = 6). Amplitude-integrated electroencephalogram (aEEG) was recorded and magnetic resonance spectroscopy (MRS) was acquired at 24 and 48 h. At 48 h, terminal deoxynucleotidyl transferase dUTP nick-end labeling (TUNEL)-positive brain cell death, microglial activation/proliferation, astrogliosis, and cleaved caspase-3 (CC3) were quantified. Hematology and plasma cytokines were serially measured.

**Results:**

Two HT piglets died. aEEG recovery, thalamic and white matter MRS lactate/*N*-acetylaspartate, and TUNEL-positive cell death were similar between groups. HT increased microglial activation in the caudate, but had no other effect on glial activation/proliferation. HT reduced CC3 overall. HT suppressed platelet count and attenuated leukocytosis. Cytokine profile was unchanged by HT.

**Conclusions:**

We did not observe protection with HT in this piglet IS-HI model based on aEEG, MRS, and immunohistochemistry. Immunosuppressive effects of HT and countering neuroinflammation by LPS may contribute to the observed lack of HT efficacy. Other immunomodulatory strategies may be more effective in IS-HI.

**Impact:**

- Acute infection/inflammation is known to exacerbate perinatal brain injury and can worsen the outcomes in neonatal encephalopathy.
- Therapeutic HT is the current standard of care for all infants with NE, but the benefit in infants with coinfection/inflammation is unknown.
- In a piglet model of inflammation (LPS)-sensitized HI, we observed no evidence of neuroprotection with cooling for 24 h, based on our primary outcome measures: aEEG, MRS Lac/NAA, and histological brain cell death.
- Additional neuroprotective agents, with beneficial immunomodulatory effects, require exploration in IS-HI models.

## Introduction

Therapeutic hypothermia (HT) is standard care for neonates with intrapartum-related neonatal encephalopathy (NE) under intensive care settings. Cooling to 33.5 °C for 72 h improves survival free from disability in the short and long term.^1,2^ However, cooling is ineffective in preventing disabilities in a proportion of infants (number needed to treat, 7 (95% confidence interval (CI) 5–10) for reduction in death or neurodisability at 18 months).^1^ It is clear that, while disability from cerebral palsy (CP) in NE survivors reduces from 35%^1^ to as low as 16%^3^ with HT, some level of intellectual impairment may remain even in the absence of CP.^4^ Attenuated efficacy of HT may be due to severity of illness, timing of insult in relation to initiation of HT, or sensitization with coexisting infection/inflammation.

In preclinical laboratory studies demonstrating HT efficacy, NE is typically modeled with hypoxic–ischemic (HI) brain injury. However, in the clinical setting, the etiology of NE is multifactorial, with contributions from antenatal and placental pathology,^5^ genetic susceptibility,^6^ and perinatal infection/inflammation^7^ in addition to sentinel HI events.^8^ Preclinical^7,9,10^ and clinical^11^ studies suggest that acute infection/inflammation sensitizes the brain to subsequent HI, lowering the injury threshold and worsening outcome.^7,12^ Mortality amongst infants with combined NE and infection is increased compared with NE alone.^13^ We have recently demonstrated an increase in mortality and brain cell death in *Escherichia coli* (*E. coli*) LPS-sensitized hypoxic brain injury in the piglet model, compared with either *E. coli* LPS or hypoxia alone.^10^ These data concur with studies of small animal models of inflammation-sensitized HI (IS-HI).^9,14,15^ Given the increase in the severity of brain injury and mortality, effective therapeutic interventions are urgently needed for infants with IS-HI.

The benefit and safety of cooling infants with IS-HI are unclear. HT induces alterations in leukocyte number, microglial cell^16^ activation,^17^ cytokine/chemokine profiles,^18^ and hemodynamics;^19^ it is plausible that immunomodulatory effects of HT may not be beneficial in the context of inflammation sensitization. In a small prospective study of placental histology relative to magnetic resonance imaging (MRI) outcomes, HT was less protective for infants with NE where placental histology showed chorioamnionitis.^20^ In low-resource settings, where rates of IS-HI are high, HT may be less effective, and potentially harmful.^21,22^ In addition, HT has been shown to be deleterious in the presence of meningitis^23^ and sepsis^24^ in adult clinical studies. These observations emphasize the critical need for preclinical animal models that assess the safety and efficacy of HT and other neuroprotective agents in IS-HI. Rodent IS-HI models suggest variable, pathogen-dependent, efficacy of HT.^14,15,25^ This has not previously been examined in a large animal model.

The aim of this study was to assess the safety and efficacy of HT in an *E. coli* LPS-sensitized HI piglet model. The piglet model has strong similarities to newborn infants with NE in terms of the evolution and pattern of injury after HI and neuropathology.^26,27^ We hypothesized that HT would not provide neuroprotection in IS-HI based on our primary outcome measures: (i) amplitude-integrated electroencephalogram (aEEG) background activity recovery over 48 h; (ii) proton (^1^H) magnetic resonance spectroscopy (MRS) lactate/*N*-acetylaspartate ratio (Lac/NAA) at 48 h in the subcortical white matter and thalamus; and (iii) terminal deoxynucleotidyl transferase dUTP nick-end labeling (TUNEL) immunolabelling of histological cell death over the whole-brain and eight brain regions at 48 h after HI.

## Methods

This study was conducted in accordance with the UK Home Office Guidelines [Animals (Scientific procedures) Act, 1986] and complies with ARRIVE guidelines. The Ethics Committee of University College London approved the study.

### Sample size calculation

Using previous data from our piglet study of LPS plus hypoxia (geometric least-squares mean standard deviation of 17), we estimated that six piglets per intervention group were required to detect a difference in TUNEL-positive cells of >30 cells/mm^2^, using a significance threshold of 5 and 80% power.

### Animal experiments and surgical preparation

Male large white piglets aged ≤36 h were sedated with intramuscular midazolam (150 μg) and anesthetized with 2–3% (v/v) isoflurane. Piglets underwent tracheostomy and mechanical ventilation (SLE 2000 Infant Ventilator, Surrey, UK). The bilateral common carotid arteries were surgically isolated and encircled by inflatable occluders (OC2A, In Vivo Metric). A 2-French central venous catheter (Vygon, Swindon, UK) was inserted into the axillary or brachial vein for later infusion of LPS. Umbilical catheters were sited; a 4-French double-lumen umbilical venous catheter (Vygon) was used for maintenance fluids (10% dextrose, 60 ml/kg/day, reduced to 40 ml/kg/day post HI), fentanyl (3 μg/kg/h), and antibiotics (benzylpenicillin 50 mg/kg every 12 h and gentamicin 2.5 mg/kg every 24 h); and a 2.5-French umbilical arterial catheter (Vygon) for intermittent blood sampling (Abbot Laboratories, UK) and continuous monitoring of heart rate and mean arterial blood pressure (MABP). Arterial catheters were maintained with 0.3 ml/h heparinized saline (1 IU/ml in 0.9% saline solution). Core (rectal) temperature was maintained in the normothermic range during surgery using a radiant warmer. The duration of surgery was 1–2 h.

Following surgery, piglets were positioned prone within a bespoke MRI-compatible transport incubator. Intensive care support and physiological monitoring (SA Instruments, London, UK) was continued throughout the study. Ventilation was adjusted to maintain partial pressure of oxygen (PaO_2_) and carbon dioxide (PaCO_2_) at 8–13 and 4.5–6.5 kPa, respectively. MABP was supported with crystalloid (0.9% saline, Baxter, 10 ml/kg bolus) and inotropes (dopamine 5–25 μg/kg/min, dobutamine 5–20 μg/kg/min, noradrenaline 0.1–1 μg/kg/min, adrenaline 0.1–1 μg/kg/min) to target MABP >35 mm Hg. Anesthesia was maintained throughout with fentanyl (3 μg/kg/h infusion) and isoflurane (2–3% (v/v)). Hyperkalemia (potassium > 7.0 mmol/l) was treated with 4 μg/kg intravenous salbutamol over 10 min.

### EEG acquisition

Multichannel six-lead EEG (Nicolet, CareFusion, Madison, WI) was commenced immediately post surgery and maintained throughout the study. aEEG recordings were retrospectively scored hourly using the pattern classification.^28^ Flat trace was assigned a score of 0; continuous low voltage 1; burst suppression 2; discontinuous normal voltage 3; and continuous normal voltage 4. Scores were averaged every 6 h. Electrographic seizures were treated with phenobarbitone, initially 20 mg/kg, followed by 10 mg/kg doses for subsequent seizures, up to a maximum 40 mg/kg.

### Broadband near-infrared spectroscopy

A broadband near-infrared spectroscopy (bNIRS) system (Mini-CYRIL, Cytochrome Research Instrument and Application System, UCL, UK) was used to measure the concentration of oxidized cytochrome *c* oxidase (oxCCO).

### LPS administration

Baseline physiological, bNIRS, and EEG data were captured prior to administration of LPS. All piglets received a bolus of 2 μg/kg LPS (*E. coli*-derived, Sigma LPS O55:B5, St. Louis, MO), followed by a continuous infusion of 1 μg/kg/h LPS for the duration of the experiment (total 52 h LPS).

### Transient HI

Four hours after the LPS bolus, the carotid occluders were inflated and the fraction of inspired oxygen (FiO_2_) was reduced to induce transient HI. FiO_2_ was decreased sequentially to 6% over the first 3 min and maintained. The insult was titrated using continuous MABP, EEG, and bNIR monitoring, and arterial blood gas was measured at 5-min intervals. Target insult parameters included MABP between 26 and 30 mm Hg, a 3-fold reduction in oxCCO from baseline, a sustained isoelectric EEG, and metabolic acidosis (pH < 7.3, lactate > 10). FiO_2_ was liberalized in the event of MABP and oxCCO values beyond the target range. The duration of insult was anticipated to be 20 min. At the end of the insult, occluders were deflated, FiO_2_ normalized to 21%, and the animal resuscitated as necessary.

### Experimental groups: temperature management

**Fig. 1:**
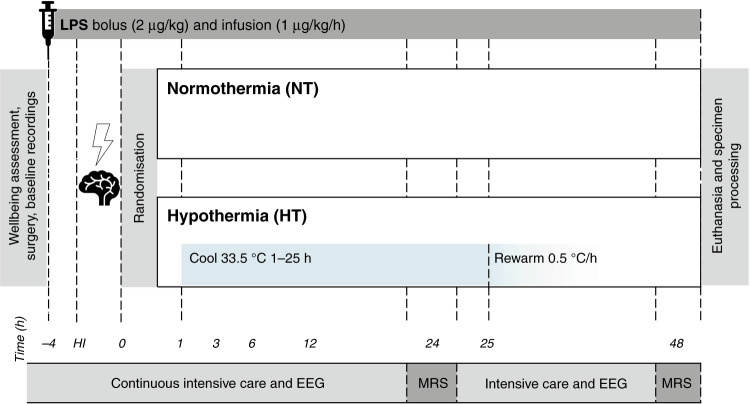
Study timeline. Following baseline recordings and surgery, piglets received a 2 µg/kg LPS bolus, followed by 1 µg/kg/h LPS infusion continued for 52 h. After 4 h of infusion, piglets underwent a transient hypoxic–ischemic (HI) insult. Animals were subsequently randomized to (i) normothermia (NT, core 38.0–39.0 °C throughout) or (ii) hypothermia (HT, 33.5 °C from 1 to 25 h post HI, then rewarmed at 0.5 °C/h). Blood samples were collected at baseline, 4 h after bolus, end HI (time 0), and at 3, 12, 24, and 48 h after HI. EEG was continuously acquired, excluding during MRS at 24 and 48 h. Piglets were maintained under meticulous intensive care for 48 h following HI, prior to euthanasia and histological specimen collection.

Following insult, piglets were randomized, using a computer-generated randomization sequence and opaque sequentially numbered envelopes, to two groups: (i) LPS, HI, and normothermia (NT) and (ii) LPS, HI, and HT (Fig. 1). NT piglets had core temperature maintained in the normothermic range (38.0–39.0 °C) throughout the study. HT piglets were actively cooled to 33.5 °C from 1 to 25 h post insult, and then rewarmed over 10 h at an incremental rate of 0.5 °C/h.^17^ HT piglets were subsequently maintained in the normothermic range until the end of the study. Temperature was maintained within target ranges using a water mattress (Tecotherm TSmed200, Inspiration Healthcare, Crawley, UK) manually adjusted according to continuous rectal temperature. The mattress was wrapped around the trunk and limbs.

### Magnetic resonance spectroscopy

MRS was acquired on a Philips Achiever 3 T (Philips Healthcare, Best, The Netherlands) scanner at 24 and 48 h after HI. ^1^H magnetic resonance spectra were acquired using a Philips 32-channel head coil. Chemical shift imaging (CSI) based on a two-dimensional point-resolved spectroscopy excitation used an 8 × 8 matrix with a voxel size of 7.5 × 7.5 mm^2^ and slab thickness of 10 mm. The repetition time was 2 s and the echo time was 288 ms. ^1^H CSI data was processed using spectroscopy analysis software, TARQUIN,^29^ yielding signal amplitudes for NAA and lactate. Two regions were selected for the final analysis: a single voxel over the dorsal right subcortical white matter at the centrum semi-ovale level and the sum of two voxels over the lateral thalami.

### Hematology and cytokine analysis

Arterial blood was collected at baseline, 4 h after LPS bolus, at the end of HI, and at 3, 12, 24, and 48 h post HI. Cerebrospinal fluid (CSF) was obtained by a lumbar puncture at baseline and at 48 h. Hematology samples were stored at 4 °C for a maximum of 72 h prior to complete blood film examination (Royal Veterinary College Diagnostic Laboratory, Hawkshead Campus, UK). Hemoglobin (Hb), total white cell (WBC), neutrophil, lymphocyte, monocyte, and platelet count were analyzed. Serum and CSF samples were frozen (−20 °C) for later protein analysis. Multiplex porcine enzyme-linked immunosorbent assay (ELISA) was undertaken to quantify interleukin-1β (IL-1β), IL-6, IL-8, and tumor necrosis factor-α (TNFα) (Q-Plex Porcine Cytokine—High sensitivity (4-Plex), ref.: 119149PC, Tebu-Bio, France), while a porcine ELISA was utilized for IL-10 (Porcine IL-10 ELISA Kit (Cloud-Clone Corp., ref. SEA056Po), Tebu-Bio, France).

### Brain histology

Forty-eight hours after HI, piglets were euthanized and histological brain specimens were obtained and prepared as previously described.^10^ Cell death was quantified by nuclear DNA fragmentation using histochemistry with TUNEL. Glial activation was quantified using the presence of astrocyte glial fibrillary acidic protein (GFAP) and microglial ionized calcium-binding adaptor molecule 1 (IBA1) immunoreactivity. Cleaved caspase-3 (CC3) immunoreactivity was quantified. Histological analysis was performed by investigators blind to the treatment group. Eight brain regions were assessed (cingulate cortex, sensorimotor cortex, hippocampus, periventricular white matter, internal capsule, caudate nucleus, putamen, and thalamus). For each region, TUNEL-positive nuclei were counted in six fields (three per section, at ×40 magnification, with an area of 0.066 mm^2^) and the average converted into counts per mm^2^. IBA1-positive cell body count was similarly performed. In addition, IBA1 ramification index was calculated in six fields per region, at ×40 magnification, using a 0.049 mm × 0.049 mm square grid. The microglial ramification index was calculated as (*B*^2^/*C*), where *C* is the number of cell bodies within the grid, and *B* is the number of branches crossing the three horizontal and three vertical gridlines. CC3 immunoreactive cells were counted in four fields per region (at ×20 magnification with an area of 0.164 mm^2^) and the average converted into counts per mm^2^. To quantify the GFAP immunoreactivity optical luminosity values were calculated by deducting mean brightness values of the tissue (four fields per region at ×20 magnification) from the mean brightness of the blank slide.

### Statistical methods

Non-normally distributed data were log-transformed prior to analysis. Using SAS JMP® Pro v14.0.0, analysis of variance models were fitted to give estimates of expected treatment group mean values and difference between means.  For TUNEL and histology measurements, the effects of treatment, region, and the interaction between them were fitted to the mean result for each subject averaged across replicate measurements within each region. For EEG, MRS, cytokines, and hematology, a model with terms for treatment, time interval (as a factor) and the interaction between them, and a random subject effect to take into account the repeated-measures structure was fitted to the results for each subject averaged across each time interval. Using the model, a comparison to baseline was calculated for hematology values. GraphPad Prism® v8 was used to calculate Pearson’s correlation between TUNEL and CC3 cell count. Cytokine change from baseline was calculated using Wilcoxon’s matched-pairs signed-rank test. Using IBM SPSS® v22, physiological data and clinical outcomes were analyzed using *t* test or Fisher’s exact test as appropriate.

## Results

### Baseline characteristics and physiological parameters

Piglet weight ranged from 1800 to 2250 g. Physiological parameters are shown in Table 1. At baseline groups were similar. During the time epochs 1–25 and 25–35 h, which encompass cooling and rewarming, the HT subjects had lower core temperatures as expected (*p* < 0.001 and 0.002, respectively).

**Table 1:** Physiological observations and blood gas results throughout the experiment.

	Normothermia		Hypothermia	
	Mean	(SD)	Mean	(SD)
Weight (g)	2008	(132)	2075	(161)
Rectal temp ( °C)				
Baseline	37.9	(0.8)	37.6	(0.4)
4 h LPS	38.6	(0.3)	38.6	(0.3)
End of insult	38.1	(0.5)	38.1	(0.3)
1–25 h	38.4	(0.1)	34.0	(0.5)**
25–35 h	38.7	(0.1)	36.5	(1.3)**
35–48 h	38.4	(0.2)	38.2	(0.2)
Heart rate (b.p.m.)				
Baseline	169.1	(26.0)	151.7	(11.9)
4 h LPS	192.9	(15.0)	185.0	(12.6)
End of insult	237.3	(15.8)	225.6	(12.9)
1–25 h	234.9	(20.5)	170.9	(24.8)**
25–35 h	210.0	(25.2)	180.0	(8.7)*
35–48 h	185.2	(21.7)	173.0	(13.1)
MABP (mm Hg)				
Baseline	51.1	(4.3)	52.1	(1.4)
4 h LPS	53.1	(3.7)	54.1	(5.8)
End of insult	47.3	(6.3)	43.7	(5.6)
1–25 h	45.7	(5.4)	42.7	(9.8)
25–35 h	55.0	(5.2)	42.8	(15.0)
35–48 h	49.5	(2.9)	54.8	(4.5)
pH				
Baseline	7.48	(0.19)	7.57	(0.10)
4 h LPS	7.44	(0.10)	7.45	(0.07)
End of insult	7.22	(0.11)	7.25	(0.13)
12 h	7.49	(0.07)	7.44	(0.17)
24 h	7.47	(0.03)	7.38	(0.13)
48 h	7.47	(0.04)	7.48	(0.05)
PaCO_2_ (kPa)				
Baseline	6.3	(3.4)	4.3	(0.8)
4 h LPS	5.8	(1.0)	5.6	(0.7)
End of insult	6.3	(1.8)	5.6	(0.9)
12 h	5.3	(0.6)	4.4	(1.1)
24 h	5.4	(0.7)	5.1	(1.3)
48 h	5.2	(0.6)	5.0	(0.4)
PaO_2_ (kPa)				
Baseline	18.6	(2.7)	15.0	(4.3)
4 h LPS	10.4	(1.1)	10.8	(0.9)
End of insult	3.6	(0.4)	3.7	(0.9)
12 h	13.7	(4.8)	15.5	(6.6)
24 h	12.8	(3.1)	10.7	(4.0)
48 h	15.2	(3.9)	14.6	(1.8)
BE (mmol/L)				
Baseline	8.5	(3.3)	7.7	(4.4)
4 h LPS	5.9	(3.4)	5.1	(3.4)
End of insult	−9.0	(2.9)	−8.7	(4.9)
12 h	6.8	(3.4)	−0.3	(10.5)
24 h	5.7	(2.1)	−1.7	(9.8)
48 h	4.5	(2.4)	5.4	(4.9)
Lactate (mmol/L)				
Baseline	3.6	(1.7)	2.9	(1.2)
4 h LPS	4.1	(2.0)	3.4	(1.3)
End of insult	13.1	(1.7)	11.0	(2.2)
12 h	3.0	(0.6)	6.6	(5.0)
24 h	2.3	(0.8)	6.7	(7.1)
48 h	1.1	(0.2)	1.3	(0.3)
Glucose (mmol/L)				
Baseline	4.9	(1.3)	5.2	(1.2)
4 h LPS	6.0	(2.2)	5.6	(0.5)
End of insult	8.9	(3.0)	7.9	(1.0)
12 h	6.8	(1.2)	11.5	(5.6)
24 h	6.5	(1.2)	10.3	(3.6)*
48 h	4.5	(1.2)	4.9	(0.7)
Potassium (mmol/L)				
Baseline	4.1	(0.7)	4.5	(0.4)
4 h LPS	4.5	(0.4)	5.0	(0.4)
End of insult	4.5	(0.5)	5.2	(0.8)
12 h	6.3	(0.7)	6.3	(1.0)
24 h	6.7	(0.7)	6.7	(1.4)
48 h	5.7	(1.2)	6.2	(1.3)

*MABP* mean arterial blood pressure, *BE* base excess.

*P < 0.05, **P < 0.005.

### HI insult

Insult severity, as determined by the duration of insult, MABP < 30, isoelectric EEG, area under the curve (AUC) FiO_2_, AUC oxCCO, and end HI acid–base status, was similar between groups (Table 2).

**Table 2:** HI insult severity measures.

	Normothermia		Hypothermia	
	Mean	(SD)	Mean	(SD)
Duration HI (min)	20.7	(1.4)	21.2	(2.1)
AUC FiO_2_ (%)	295.8	(20.1)	290.5	(43.2)
Duration of EEG < 5 μV (min)	15.5	(2.9)	17.7	(1.8)
Duration of MABP < 30 (min)	7.3	(2.9)	10.0	(3.8)
AUC oxCCO	146	(25)	124	(50)
End of HI arterial gas				
pH	7.22	(0.11)	7.25	(0.13)
pCO_2_ (kPa)	6.3	(1.8)	5.6	(0.9)
pO_2_ (kPa)	3.6	(0.4)	3.7	(0.9)
Base excess	−9.0	(2.9)	−8.7	(4.9)
Lactate (mmol/L)	13.1	(1.7)	11.0	(2.2)

No comparisons were *p* < 0.05

*HI* hypoxia–ischemia, *EEG* electroencephalogram, MABP mean arterial blood pressure, *oxCCO* oxidized cytochrome *c* oxidase.

### Survival and clinical illness severity

During active cooling, HT animals had a lower mean heart rate than NT animals (171 (SD 25) b.p.m. compared with 235 (SD 21) b.p.m., *p* = 0.001). Mean heart rate was also lower during rewarming for HT animals (*p* = 0.02) (Table 1). Mean MABP, inotropic support, and saline bolus requirement were not different between groups (Table 3). Mean infusion rates of all inotropes were higher in the HT group; however, confidence intervals are wide. HT had higher mean blood glucose at 24 h (10.3 (SD 3.6) mmol/L compared with 6.5 (SD 1.2) mmol/L, *p* = 0.033). Three of six NT and four of six HT subjects had hyperkalemia necessitating treatment (*p* = 0.5).

**Table 3:** Inotrope and saline bolus use throughout the experiment.

	Normothermia		Hypothermia	
	Mean	(SD)	Mean	(SD)
Dopamine (μg/kg/min)	10.1	(4.8)	15.6	(6.4)
Dobutamine (μg/kg/min)	2.8	(4.5)	4.5	(6.9)
Noradrenaline (ng/kg/min)	16	(37)	96	(149)
Adrenaline (ng/kg/min)	0	(0)	484	(811)
10 ml/kg Saline bolus (*n*)	0.7	(0.8)	0.8	(1.3)

No comparisons were *p* < 0.05.

Two of six piglets died prior to experiment completion in the HT group, compared with none in the NT group (*p* = 0.23). The two animals had refractory hypotension and worsening metabolic acidosis at the time of cardiac arrest (30 and 33 h). Both were rewarmed early due to the severity of hypotension (at 16 and 12.5 h). One had severe hyperkalemia (K^+^ > 9). Both had macroscopic necrosis to abdominal solid organs including the liver, kidneys, and bowel.

### TUNEL

**Fig. 2:**
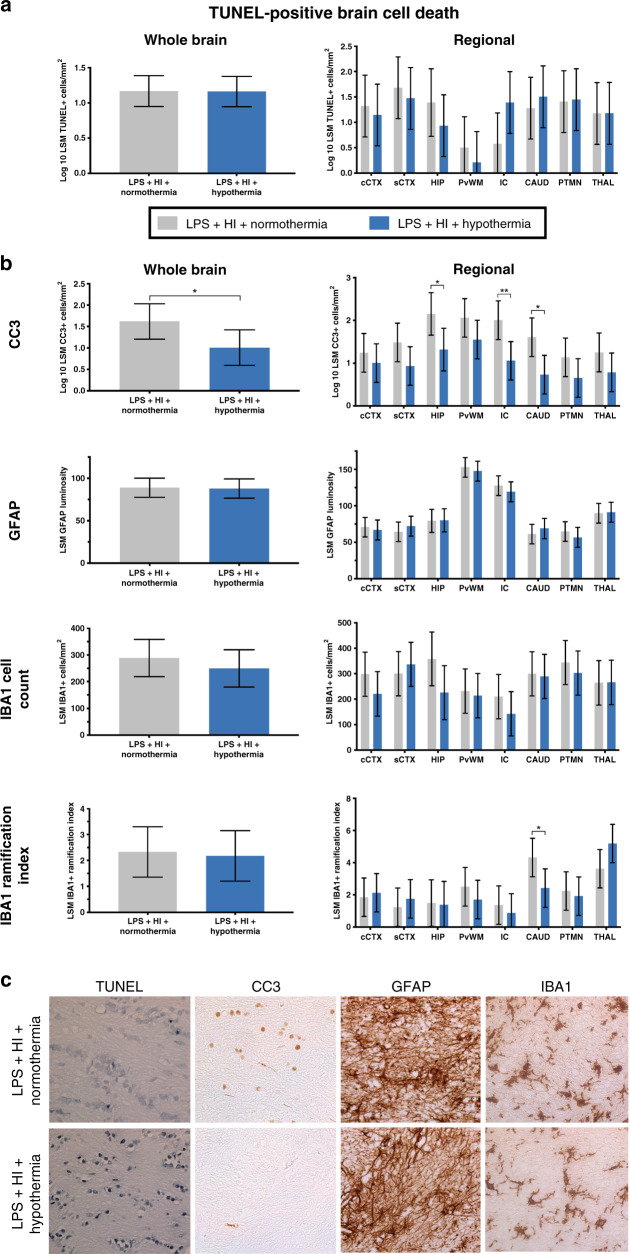
Brain histology. **a** TUNEL histology. There was no difference between whole-brain or regional estimated least-squares mean (LSM) TUNEL-positive cells per mm^2^. **b** LSM whole-brain and regional cleaved caspase-3 (CC3), GFAP, IBA1-positive cell count, and IBA1 ramification index. Hypothermia reduced CC3 throughout the brain and in the internal capsule, caudate nucleus, and hippocampus. Hypothermia did not alter astrogliosis. Cooling had no effect on the overall microglial number or activation state. There was an increase in microglial activation (lower ramification index) in the caudate with HT. **c** Representative sections for each stain (TUNEL, CC3, GFAP, and IBA1) from the internal capsule are shown at ×40 magnification. cCTX cingulate cortex, sCTX sensorimotor cortex, HIP hippocampus, PvWM periventricular white matter, IC internal capsule, CAUD caudate, PTMN putamen, THAL thalamus. Data are displayed as analyzed, on a log 10 scale. Error bars represent 95% CI. *p < 0.05, **p < 0.01.

Mean whole-brain TUNEL-positive cell counts were similar between the NT and HT groups (*p* = 0.97) (Fig. 2a). The regional assessment was also similar between groups. There was a trend towards greater histological cell death in the internal capsule for the TH group (*p* = 0.064).

### Cleaved caspase-3

HT reduced overall CC3 (Fig. 2b) compared with NT (*p* = 0.04). The regional analysis demonstrates lower CC3 in the internal capsule, caudate nucleus, and hippocampus (*p* = 0.006, 0.01, and 0.02, respectively). There was no correlation between TUNEL-positive cell count and CC3-positive cell count (*r* = −0.42, 95% CI −0.54 to −0.30).

### Glial fibrillary acidic protein

Mean whole-brain and regional GFAP luminosity was similar between the NT and HT groups (p = 0.89) (Fig. 2b).

### Ionized calcium-binding adaptor molecule 1

IBA1-positive cell count was no different between the NT and HT group throughout the whole brain (*p* = 0.41) and in each brain region (*p* ≥ 0.08) (Fig. 2b). Overall microglial activation state (ramification index) was also similar between groups (*p* = 0.81). There was an increase in microglial activation (lower ramification index) in the caudate nucleus with HT (*p* = 0.029). Microglia for both groups appeared partially activated, with enlarged cell bodies, a reduced number of processes, and thickening of the remaining processes.

Representative photomicrographs from the internal capsule area at ×40 magnification for all histological stains are shown in Fig. 2c.

### Amplitude-integrated electroencephalogram

**Fig. 3:**
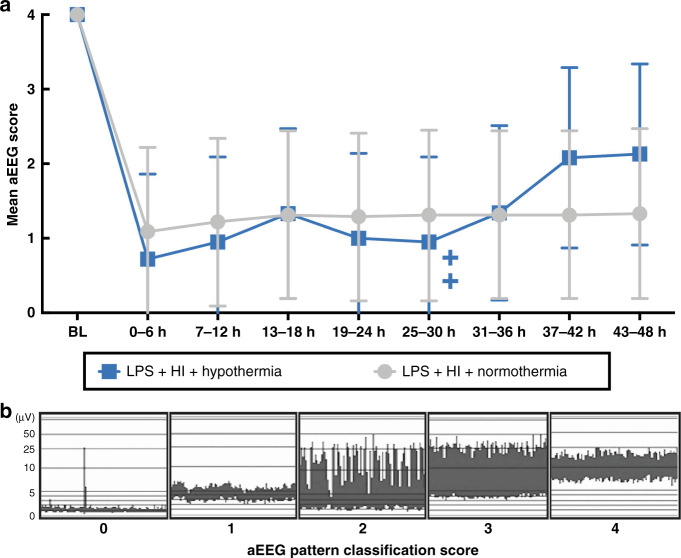
aEEG. Mean aEEG pattern classification score from baseline (BL) till 48 h, divided into 6 h time epochs (**a**) (±95% CI). There was no difference between NT and HT aEEG scores at any time. The two crosses represent the deaths of two HT piglets at 30 and 33 h. Examples of each pattern classification score are shown (**b**), where 0 = flat trace, 1 = low voltage, 2 = burst suppression, 3 = discontinuous normal voltage, and 4 = continuous normal voltage.

All subjects had a normal (score 4) aEEG at baseline (Fig. 3). The mean aEEG score for both groups was suppressed following HI and remained suppressed throughout the experiment. There was no effect of HT on aEEG recovery. Two of the six NT and one of the six HT piglets had electrographic seizures (*p* = 0.5). Seizure onset was at 12 and 15 h post HI for the two NT piglets, and at 36 h for the one HT piglet with seizures. Seizure burden was no different between groups (range 0–47 min) (*p* = 0.75). Three of the six NT and one of the six HT piglets were treated with phenobarbitone. Phenobarbitone use and dose per kg were not different between groups (*p* ≥ 0.34).

### Magnetic resonance spectroscopy

**Fig. 4:**
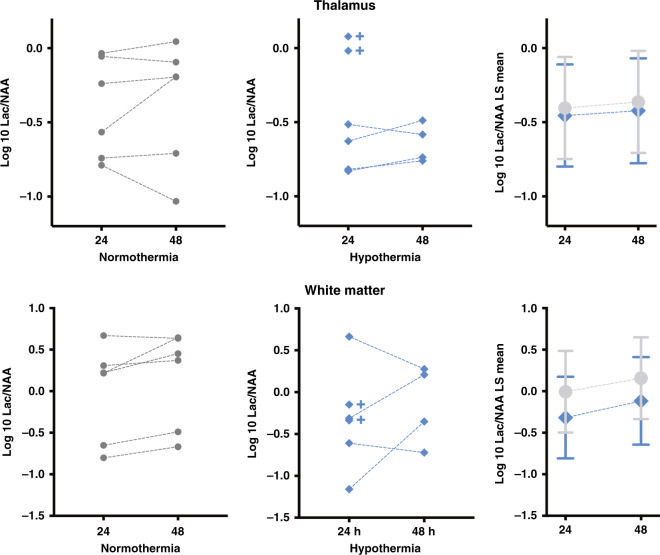
MRS. There is no effect of hypothermia on the ratio of lactate to *N*-acetylaspartate (Lac/NAA) in the thalamus or white matter at 24 or 48 h after HI. In all subjects, log 10 Lac/NAA data points are shown in the graphs on the left/center, and are summarized using log 10 least-squares (LS) means plot with 95% CI error bars in the boxes on the right. The crosses indicate the two HT piglets who died prior to the 48 h scan.

Lac/NAA was no different between the cooled and normothermic piglets at either time point, in either the white matter or thalamus (Fig. 4, *p* ≥ 0.35 for all comparisons). Note that due to early death, two of six HT subjects were not scanned at 48 h. Compared to all others, these two deceased piglets had the highest thalamic Lac/NAA at 24 h.

### Hematology

**Fig. 5:**
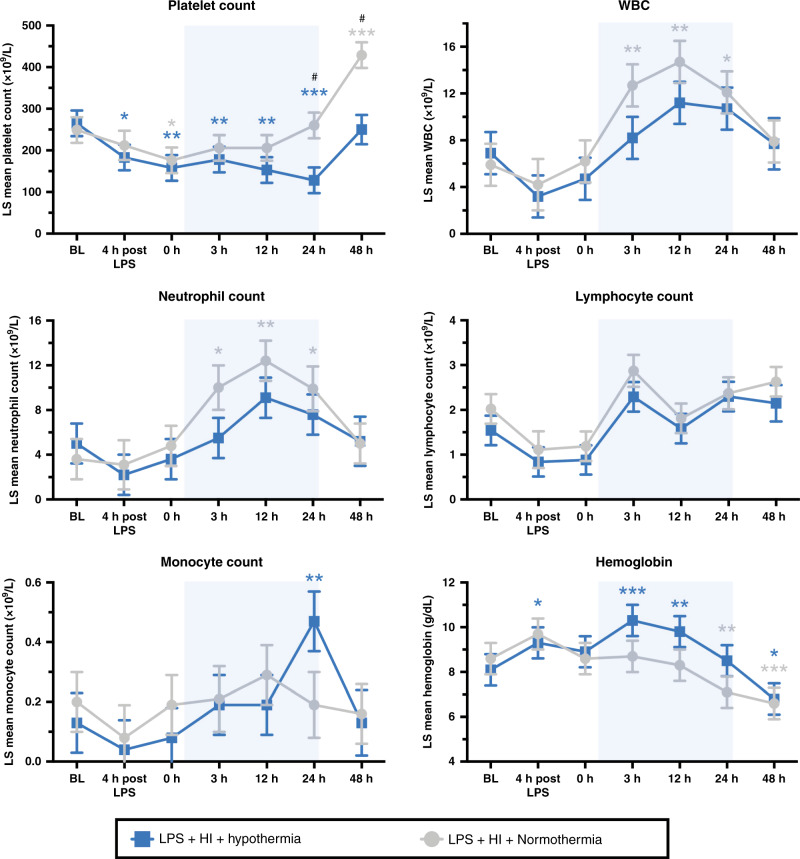
Hematology. Least-squares mean values over time for platelets, white blood cells (WBC), neutrophils, lymphocytes, monocyte counts, and hemoglobin (±SEM). Pale blue shading represents cooling. *P < 0.05, **p < 0.01, and ***p < 0.0001 for change from baseline value, with color denoting group; ^#^*p* < 0.05 for the difference at timepoint between the NT and HT group.

In both groups, platelet count fell below baseline by time 0 (*p* ≤ 0.024) (Fig. 5). NT platelet count returned to baseline levels, before a late thrombocytosis at 48 h post HI (*p* < 0.001). HT platelet count continued to fall below baseline for the duration of cooling therapy (*p* ≤ 0.007), before a later recovery to baseline at 48 h. At 24 and 48 h after HI, platelet count was suppressed in the HT group compared with NT (*p* = 0.005 and 0.001, respectively).

The WBC increased from baseline for NT subjects at 3, 12, and 24 h (*p* ≤ 0.016) (Fig. 5). This increase was attenuated in HT subjects. There was no significant difference between WBC for NT and HT animals at any single time point. Neutrophils were the predominant leukocyte present and the analysis closely mirrored that for WBC. There was no effect of cooling or time on lymphocyte number. The monocyte count was raised at 24 h in the HT group compared to baseline (*p* = 0.002). Monocytes were otherwise unaltered by HT or time.

There was no intergroup difference for Hb at any time point. NT Hb was stable and then fell below baseline from 24 h (*p* = 0.002). Hb increased from baseline in the HT group at 4 h after LPS and at 3 and 12 h after HI (*p* ≤ 0.012) (Fig. 5). At 48 h, both HT and NT Hb were below baseline (*p* ≤ 0.022).

### Cytokines

**Fig. 6:**
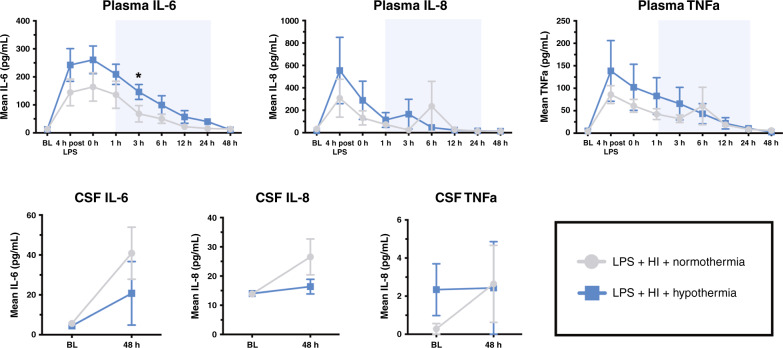
Cytokines. Mean values over time for IL-6, IL-8, and TNFα in the plasma and cerebrospinal fluid (CSF) (±SEM). Pale blue shading represents cooling. *P < 0.05 for difference at timepoint between the NT and HT group.

The pattern of IL-6, IL-8, and TNFα in the plasma and CSF is shown in Fig. 6. Plasma IL-6, IL-8, and TNFα increased from baseline pre-HI (*p* ≤ 0.0034) in response to LPS. HT plasma IL-6 was higher than NT at 3 h post HI (*p* = 0.018). There were no other differences between groups and no difference in CSF cytokines. IL-1β and IL-10 were below the limit of detection for the majority of samples and therefore were not analyzed.

## Discussion

In this piglet model of IS-HI, we did not observe protection with 24 h HT (from 1 to 25 h after HI) based on pre-defined primary outcome measures; aEEG, MRS, and histological cell death (TUNEL-positive cells). Microglial activation and glial cell proliferation were unaltered, although microglial activation was increased in the caudate nucleus with HT. Compared to NT, HT suppressed the platelet and white cell count response. Plasma and CSF cytokine response to LPS and HI was largely unchanged with HT. HT reduced CC3 throughout the brain; in our model, CC3 is a poor marker of cell death,^30^ and in this and previous studies, a regional reduction in CC3 is associated with increased TUNEL-positive cells.

These results differ from non-sensitized HI piglet models, where HT for 24 h started at 2 h after HI reduced brain injury based on TUNEL-positive cell counts^17^ and HT for 12–24 h started immediately after HI ameliorated secondary energy failure, reduced seizure activity, and improved neurological function and histopathology scores.^31,32^ In the present study, a trend towards increased TUNEL-positive cell death in the internal capsule with HT was observed. Concurring with the histological data, aEEG and ^1^H MRS were not improved by HT. Comparing NT and HT groups, there was no difference in aEEG score or seizure burden. The mean aEEG score for HT trended upwards from 31 h; this may be attributed to the loss of two subjects who had flat traces (score 0). In NE babies, a recovery of aEEG within 48 h is associated with a good outcome.^33^ ^1^H MRS Lac/NAA peak area ratios in the white matter and thalamus were not improved by HT. In non-sensitized piglet models, HT for 24 h improves MRS biomarkers, including Lac/NAA^31,34,35^ which is a robust marker of 2-year neurodevelopmental outcome in NE babies.^36^

Suppression of microglial activity is a favorable marker^7^ and a key mechanism by which HT is neuroprotective.^37,38^ Reduced microglial activation following HT is observed in non-sensitized HI models, including the pig,^17^ sheep,^38^ and rodent.^39^ In this IS-HI model, microglial number and morphology were not suppressed by HT; indeed, in the caudate nucleus, microglial morphology was indicative of a pro-inflammatory activation state with HT, possibly reflecting increased injury. Our findings concur with those from a rodent LPS-sensitized HI cooling model.^40^ The balance between activated microglia with a pro-inflammatory phenotype and those with an anti-inflammatory restorative phenotype is complex and needs further study.^7,12,41^ Astrogliosis, measured by GFAP, was not impacted by HT. In non-sensitized HI models, HT reduces astroglial activation.^39^ LPS enhances astrogliosis by stimulating the release of TNFα, IL-1α, and IL-6, mediators of astrocyte proliferation and activation.^42,43^ A failure of HT to suppress astrogliosis has also been shown in an LPS-sensitized rodent model.^40^

CC3 was reduced by HT, both overall and specifically in the internal capsule, caudate nucleus, and hippocampus. As in our previous LPS + hypoxia study^10^ and other recent piglet neuroprotection studies,^44,45^ CC3 was not associated with TUNEL-positive cell death. The use of male piglets may partly explain these data; cell death is sexually dimorphic, and in males, cell death pathways are predominantly caspase-independent.^46–48^ We may then conclude that CC3 is, therefore, a poor marker of cell death in this model. LPS is known to increase CC3 without resulting in apoptotic cell death.^10,49^ This CC3 response to LPS likely reflects the immunologic functions of caspases, including microglial and lymphocyte function, cell differentiation, and autophagy.^50^ Of note, the largest reduction in CC3 with HT was observed in the internal capsule, the same region where there was a trend towards an increase in TUNEL-positive cell death.

HI, HT, and LPS each influence the hematological response. Infection/inflammation triggers thrombocytopenia and leucocytosis in neonates^51^ and piglets.^10^ HI also stimulates leucocytosis.^10,52^ HT is known to reduce circulating platelets and WBC. A meta-analysis of HT clinical trials demonstrated an increase in thrombocytopenia (relative risk (RR) 1.21 (95% CI 1.05–1.40) with HT, and an increase in leukopenia with whole-body HT (RR 5.7 (95% CI 1.02–31.82)).^1^ Similarly, in this study, HT suppressed both platelet and leukocyte (neutrophil) count. The impact of this suppression is uncertain in the setting of IS-HI. In a mouse model of LPS-sensitized HI, neutrophils were central to injury pathways, leading to leukocyte infiltration, pro-inflammatory cytokine production, and brain atrophy.^53^ By contrast, an increase in circulating neutrophils, and later lymphocytes, are necessary components of the innate and adaptive immune response to infectious stimuli. Leukopenic “immune paralysis” has been demonstrated in neonates undergoing HT and was associated with poorer outcome.^16^ Thus, alterations in immune cell number may be an important factor in the efficacy and safety of TH in IS-HI.

Many immune cells are functionally heat-sensitive^54^ and a key mechanism of HT neuroprotection is inhibition of the pro-inflammatory cascade.^18,55,56^ In a cohort of babies with NE, HT infants had reduced serum IL-6 and IL-4, and increased vascular endothelial growth factor (VEGF) compared with NT infants.^57^ In the same cohort, there was a negative correlation between the duration of cooling prior to sampling, and levels of IL-6, IL-10, TNFα, and interferon-gamma (IFNγ).^57^ In another cohort, HT lowered IL-6, and increased IL-10 at 48 h.^58^ Conversely, in a phase II cooling trial, cooled infants had a biphasic pattern of elevated IL-6, IL-8, IL-10, and chemokine (C-C motif) ligand 2 in comparison with NT infants^18^ although another prospective cohort showed serial cytokine values (IL-1, IL-6, IL-8, TNFα, IFNγ, and VEGF) were unaffected by HT.^59^ In our study, plasma and CSF cytokine profiles were not influenced by HT. IL-6, IL-8, and TNFα increased from baseline in response to LPS. IL-6 was higher at 3 h post HI in HT compared to NT. The inability of HT to suppress pro-inflammatory cytokine production in IS-HI may contribute to the lack of cooling effect. Indeed, in one cooling cohort, neonates that failed to downmodulate pro-inflammatory cytokines in response to HT had poorer outcome.^18^

Mortality in HT was 33% (two deaths), compared with no deaths in NT. Neonates with both infection/inflammation and an intrapartum hypoxic event are at increased risk of mortality; in a large meta-analysis of cooled and non-cooled infants, mortality amongst infants with both group B Streptococcus sepsis and NE was higher than for infants with NE alone (RR 2.07 (95% CI 1.47–2.91)).^13^ In our previous normothermic piglet study, the combination of LPS + hypoxia increased mortality compared with hypoxia alone.^10^ In this study, the two cardiac arrests were preceded by refractory hypotension, which may have been contributed to by HT-induced bradycardia. Indeed, in a meta-analysis of cooling trials, HT increased sinus bradycardia, although there was no increase in hypotension or major arrhythmias.^1^ The HT group was hyperglycemic during the cooling phase. HT reduces insulin secretion and increases insulin resistance in preclinical models and adult HT studies.^60^ Deranged postnatal glycemic control is an important risk factor for the adverse outcome as seen in the Coolcap study^61^ and is associated with specific patterns of brain injury in NE.^62^ Two piglets in the HT group had macroscopic abdominal organ necrosis, including the bowel, at post mortem.

There are limitations to this study. By using *E. coli* LPS to stimulate inflammation, we modeled only gram-negative perinatal infection. Rodent models of IS-HI, comparing LPS with PAM3CSK4 (a toll-like receptor (TLR) 2 agonist, which simulates gram-positive infection), demonstrate the differences in temperature, cytokine expression, CC3, and glial cell response.^40^ Importantly, the efficacy of HT was pathogen-dependent; HT was neuroprotective with PAM3CSK4 IS-HI, but not with LPS IS-HI.^14,15,25^ Two of six piglets in the HT group were rewarmed early due to refractory hypotension, consistent with clinical practice. These two piglets died prematurely, reducing the available outcome data at later time points. With reduced numbers, the study may have been underpowered to demonstrate differences in later outcome measures, including aEEG and MRS. Histological outcomes measures were available for all 12 subjects, including the two piglets that died prematurely. Only male piglets were used to reduce intergroup variation. We were therefore not able to examine the impact of sex on outcome.^46^ Future studies using this model of IS-HI will be conducted using both male and female piglets. The porcine immune system is an ideal model for immunology research, resembling humans by >80%. However, there are differences, for example, piglets are born without transplacental antibodies in contrast to human neonates.^63^ This impacts interpretation of the immunomodulatory effects of HT. The insult in this study was a combined carotid artery occlusion with hypoxia rather than a pure hypoxia model where we observed significant systemic injury.^10^

HT was undertaken for 24 h (from 1 to 25 h) in the present study, a duration that is protective in our non-sensitized piglet HI model (when HT commencement was delayed to 2 h)^17^ and allows time for gradual rewarming and subsequent neuroimaging under normothermic conditions within the timeframe of our established 48 h model.^64,65^ HT maintained for ≤24 h is neuroprotective in rodent and porcine models of non-sensitized HI.^17,31,32^ The closer to HI that HT is commenced in these models, the greater the neuroprotection and the shorter the duration of cooling required.^37^ However, optimal cooling duration, based on preclinical^66,67^ and clinical studies,^68^ is 72 h. In the fetal lamb, 72 h HT (from 3 to 75 h) provided maximal neuroprotection following HI compared with either 48 or 120 h HT, with regards to EEG and histological outcome measures,^66,69^ with the rate of rewarming of lesser importance.^70^ Thus, we cannot exclude the possibility of a different effect of 72 versus 24 h cooling in IS-HI.

In this IS-HI model, both LPS and HI stimulate systemic multi-organ inflammatory responses and injury. Plasma cytokines were 1–2 orders of magnitude higher than CSF cytokines, suggesting predominant peripheral immune cell response in this model. HT effects on the peripheral immune response may have impacted upon brain injury. Whole-body cooling was undertaken in this study, consistent with clinical care. Selective head cooling may have a differential effect on whole-body cooling, through avoidance of systemic HT immunomodulation.

Our results, suggesting no protection with HT in IS-HI, may be related to (i) failure of HT to inhibit microglial activation, astrogliosis, and inflammatory cytokine production; (ii) attenuation of neutrophil numbers by HT; (iii) secondary brain injury from hemodynamic instability; and (iv) abnormal glycemic control. Following HI, HT inhibits both protective and damaging cellular pathways; in IS-HI, the balance of inhibition may be detrimental. Given the dual role of the immune response in both contributing to and preventing injury, and the complex mechanisms underpinning the evolution of HI injury, therapies targeting immunomodulation, rather than inflammation suppression, require exploration.^7,71^

Novel neuroprotective therapies require exploration in IS-HI models. In experimental rodent models of IS-HI, promising therapies include melatonin,^72^ *N*-acetylcysteine,^73^ hydrocortisone,^74^ and inflammatory cascade inhibitors/antagonists of IL-1 receptor,^75^ nuclear factor kappa beta,^76^ plasminogen activator protein-1,^77^ and histone deacetylase.^78^ Other possible therapies include erythropoietin,^79,80^ mesenchymal stem cells (MSCs),^81^ TLR modulators,^71,82^ cyclooxygenase-2 inhibitors,^83^ and immunomodulatory antibiotics such as azithromycin^84^ and minocycline.^85^ It will be important to assess therapies with and without HT; of concern is the negated benefit, or even exacerbation of injury, observed with therapies such as IL-1 receptor antagonists^86^ and MSCs^87^ when combined with HT.

Unlike the protection seen previously with 24 h HT (from 2 to 26 h) in the piglet HI model,^17^ we did not observe protection with 24 h HT (from 1 to 25 h) in a model of IS-HI. Our observation was based on EEG, MRS, and TUNEL-positive cell death. The reduced efficacy of HT, in the context of IS-HI, may relate to the inability of HT to alter glial cell function and pro-inflammatory cytokine profile as well as systemic effects of hyperglycemia and hemodynamic instability. Hypothermic suppression of leukocytes, platelets, and CC3 may be detrimental to systemic inflammatory illness. Novel neuroprotective agents with immunomodulatory properties require exploration in IS-HI models.

## References

[1] Jacobs, S. E. et al. Cooling for newborns with hypoxic ischaemic encephalopathy. *Cochrane Database of Systematic Reviews*, CD003311. 10.1002/14651858.CD003311.pub3 (2013). 10.1002/14651858.CD003311.pub3 PMC7003568 23440789

[2] Shankaran S (2012). Childhood outcomes after hypothermia for neonatal encephalopathy. N. Engl. J. Med..

[3] Jary S, Smit E, Liu X, Cowan FM, Thoresen M (2015). Less severe cerebral palsy outcomes in infants treated with therapeutic hypothermia. Acta Paediatr..

[4] Lee-Kelland R (2020). School-age outcomes of children without cerebral palsy cooled for neonatal hypoxic-ischaemic encephalopathy in 2008-2010. Arch. Dis. Child. Fetal Neonatal Ed..

[5] Badawi N (1998). Antepartum risk factors for newborn encephalopathy: the Western Australian case-control study. BMJ.

[6] MacLennan AH, Thompson SC, Gecz J (2015). Cerebral palsy: causes, pathways, and the role of genetic variants. Am. J. Obstet. Gynecol..

[7] Hagberg H (2015). The role of inflammation in perinatal brain injury. Nat. Rev. Neurol..

[8] Nelson KB (2012). Antecedents of neonatal encephalopathy in the Vermont Oxford Network Encephalopathy Registry. Pediatrics.

[9] Eklind S (2001). Bacterial endotoxin sensitizes the immature brain to hypoxic-ischaemia injury. Eur. J. Neurosci..

[10] Martinello KA (2019). Acute LPS sensitization and continuous infusion exacerbates hypoxic brain injury in a piglet model of neonatal encephalopathy. Sci. Rep..

[11] Nelson KB, Penn AA (2015). Is infection a factor in neonatal encephalopathy?. Arch. Dis. Child. Fetal Neonatal Ed..

[12] Bhalala US, Koehler RC, Kannan S (2014). Neuroinflammation and neuroimmune dysregulation after acute hypoxic-ischemic injury of developing brain. Front. Pediatr..

[13] Tann CJ (2017). Neonatal encephalopathy with Group B Streptococcal disease worldwide: systematic review, investigator group datasets, and meta-analysis. Clin. Infect. Dis..

[14] Osredkar D (2014). Hypothermia is not neuroprotective after infection-sensitized neonatal hypoxic-ischemic brain injury. Resuscitation.

[15] Falck M (2017). Hypothermic neuronal rescue from infection-sensitised hypoxic-ischaemic brain injury is pathogen dependent. Dev. Neurosci..

[16] Jenkins DD (2013). Altered circulating leukocytes and their chemokines in a clinical trial of therapeutic hypothermia for neonatal hypoxic ischemic encephalopathy. Pediatr. Crit. Care Med..

[17] Alonso-Alconada D (2015). Brain cell death is reduced with cooling by 3.5 degrees C to 5 degrees C but increased with cooling by 8.5 degrees C in a piglet asphyxia model. Stroke.

[18] Jenkins DD (2012). Serum cytokines in a clinical trial of hypothermia for neonatal hypoxic-ischemic encephalopathy. J. Cereb. Blood Flow Metab..

[19] Armstrong K, Franklin O, Sweetman D, Molloy EJ (2012). Cardiovascular dysfunction in infants with neonatal encephalopathy. Arch. Dis. Child.

[20] Wintermark P, Boyd T, Gregas MC, Labrecque M, Hansen A (2010). Placental pathology in asphyxiated newborns meeting the criteria for therapeutic hypothermia. Am. J. Obstet. Gynecol..

[21] Pauliah SS, Shankaran S, Wade A, Cady EB, Thayyil S (2013). Therapeutic hypothermia for neonatal encephalopathy in low- and middle-income countries: a systematic review and meta-analysis. PLoS ONE.

[22] Robertson NJ (2008). Therapeutic hypothermia for birth asphyxia in low-resource settings: a pilot randomised controlled trial. Lancet.

[23] Mourvillier B (2013). Induced hypothermia in severe bacterial meningitis: a randomized clinical trial. JAMA.

[24] Itenov TS (2018). Induced hypothermia in patients with septic shock and respiratory failure (CASS): a randomised, controlled, open-label trial. Lancet Respir. Med..

[25] Falck M (2018). Hypothermia is neuroprotective after severe hypoxic-ischaemic brain injury in neonatal rats pre-exposed to PAM3CSK4. Dev. Neurosci..

[26] Azzopardi D (1989). Prognosis of newborn infants with hypoxic-ischemic brain injury assessed by phosphorus magnetic resonance spectroscopy. Pediatr. Res..

[27] Lorek A (1994). Delayed (“secondary”) cerebral energy failure after acute hypoxia-ischemia in the newborn piglet: continuous 48-hour studies by phosphorus magnetic resonance spectroscopy. Pediatr. Res..

[28] de Vries LS, Hellstrom-Westas L (2005). Role of cerebral function monitoring in the newborn. Arch. Dis. Child. Fetal Neonatal Ed..

[29] Wilson M, Reynolds G, Kauppinen RA, Arvanitis TN, Peet AC (2011). A constrained least-squares approach to the automated quantitation of in vivo (1)H magnetic resonance spectroscopy data. Magn. Reson. Med..

[30] Pang R (2020). Proton magnetic resonance spectroscopy lactate/N-acetylaspartate within 48 h predicts cell death following varied neuroprotective interventions in a piglet model of hypoxia–ischemia with and without inflammation-sensitization. Front. Neurol..

[31] Thoresen M (1995). Mild hypothermia after severe transient hypoxia-ischemia ameliorates delayed cerebral energy failure in the newborn piglet. Pediatr. Res..

[32] Tooley JR, Satas S, Porter H, Silver IA, Thoresen M (2003). Head cooling with mild systemic hypothermia in anesthetized piglets is neuroprotective. Ann. Neurol..

[33] Thoresen M, Hellstrom-Westas L, Liu X, de Vries LS (2010). Effect of hypothermia on amplitude-integrated electroencephalogram in infants with asphyxia. Pediatrics.

[34] Huun MU (2018). DHA and therapeutic hypothermia in a short-term follow-up piglet model of hypoxia-ischemia: effects on H+MRS biomarkers. PLoS ONE.

[35] Rocha-Ferreira E (2017). Systemic pro-inflammatory cytokine status following therapeutic hypothermia in a piglet hypoxia-ischemia model. J. Neuroinflamm..

[36] Mitra S (2018). Proton magnetic resonance spectroscopy lactate/N-acetylaspartate within 2 weeks of birth accurately predicts 2-year motor, cognitive and language outcomes in neonatal encephalopathy after therapeutic hypothermia. Arch.Dis. Child. Fetal Neonatal Ed..

[37] Gunn AJ (2017). Therapeutic hypothermia translates from ancient history in to practice. Pediatr. Res..

[38] Wassink G, Gunn ER, Drury PP, Bennet L, Gunn AJ (2014). The mechanisms and treatment of asphyxial encephalopathy. Front. Neurosci..

[39] Rocha-Ferreira E, Vincent A, Bright S, Peebles DM, Hristova M (2018). The duration of hypothermia affects short-term neuroprotection in a mouse model of neonatal hypoxic ischaemic injury. PLoS ONE.

[40] Osredkar D (2015). Hypothermia does not reverse cellular responses caused by lipopolysaccharide in neonatal hypoxic-ischaemic brain injury. Dev. Neurosci..

[41] Chhor V (2013). Characterization of phenotype markers and neuronotoxic potential of polarised primary microglia in vitro. Brain Behav. Immun..

[42] Girard S (2012). Postnatal administration of IL-1Ra exerts neuroprotective effects following perinatal inflammation and/or hypoxic-ischemic injuries. Brain Behav. Immun..

[43] Selmaj KW, Farooq M, Norton WT, Raine CS, Brosnan CF (1990). Proliferation of astrocytes in vitro in response to cytokines. A primary role for tumor necrosis factor. J. Immunol..

[44] Lingam I (2019). Short-term effects of early initiation of magnesium infusion combined with cooling after hypoxia-ischemia in term piglets. Pediatr. Res..

[45] Robertson NJ (2018). Melatonin as an adjunct to therapeutic hypothermia in a piglet model of neonatal encephalopathy: a translational study. Neurobiol. Dis..

[46] Charriaut-Marlangue C, Besson VC, Baud O (2017). Sexually dimorphic outcomes after neonatal stroke and hypoxia-ischemia. Int. J. Mol. Sci..

[47] Zhu C (2006). Different apoptotic mechanisms are activated in male and female brains after neonatal hypoxia-ischaemia. J. Neurochem..

[48] Hagberg H (2004). PARP-1 gene disruption in mice preferentially protects males from perinatal brain injury. J. Neurochem..

[49] Burguillos MA (2011). Caspase signalling controls microglia activation and neurotoxicity. Nature.

[50] Abraham MC, Shaham S (2004). Death without caspases, caspases without death. Trends Cell Biol..

[51] Sola-Visner M, Sallmon H, Brown R (2009). New insights in the mechansims of non-immune thrombocytopenia in neonates. Semin. Perinatol..

[52] Koreti S, Eske GS, Gaur A, Gupta A (2018). Study of hematological parameters among newborns with perinatal asphyxia. Pediatr. Rev..

[53] Yao H-W, Kuan C-Y (2018). Early neutrophil depletion reduces inflammation-sensitized hypoxic-ischemic brain injury in mouse neonates. J. Immunol..

[54] Evans SS, Repasky EA, Fisher DT (2015). Fever and the thermal regulation of immunity: the immune system feels the heat. Nat. Rev. Immunol..

[55] Kimura A, Sakurada S, Ohkuni H, Todome Y, Kurata K (2002). Moderate hypothermia delays proinflammatory cytokine production of human peripheral blood mononuclear cells. Crit. Care Med..

[56] Nakamura T, Yamada S, Yoshioka T (2013). Brain hypothermic therapy dramatically decreases elevated blood concentrations of high mobility group box 1 in neonates with hypoxic-ischemic encephalopathy. Dis. Markers.

[57] Roka A (2013). Changes in serum cytokine and cortisol levels in normothermic and hypothermic term neonates after perinatal asphyxia. Inflamm. Res..

[58] Moon CJ, Youn YA, Yum SK, Sung IK (2016). Cytokine changes in newborns with therapeutic hypothermia after hypoxic ischemic encephalopathy. J. Perinatol..

[59] Chalak LF (2014). Biomarkers for severity of neonatal hypoxic-ischemic encephalopathy and outcomes in newborns receiving hypothermia therapy. J. Pediatr..

[60] Haase KK, Grelle JL, Khasawneh FA, Ike C (2017). Variability in glycemic control with temperature transitions during therapeutic hypothermia. Crit. Care Res. Pract..

[61] Basu SK (2016). Hypoglycaemia and hyperglycaemia are associated with unfavourable outcome in infants with hypoxic ischaemic encephalopathy: a post hoc analysis of the CoolCap Study. Arch. Dis. Child. Fetal Neonatal Ed..

[62] Basu SK (2018). Early glycemic profile is associated with brain injury patterns on magnetic resonance imaging in hypoxic ischemic encephalopathy. J. Pediatr..

[63] Pabst R (2020). The pig as a model for immunology research. Cell Tissue Res..

[64] Robertson NJ (2019). Melatonin as an adjunct to therapeutic hypothermia in a piglet model of neonatal encephalopathy: a translational study. Neurobiol. Dis..

[65] Robertson NJ (2020). High-dose melatonin and ethanol excipient combined with therapeutic hypothermia in a newborn piglet asphyxia model. Sci. Rep..

[66] Davidson JO (2015). How long is too long for cerebral cooling after ischemia in fetal sheep?. J. Cereb. Blood Flow Metab..

[67] Davidson JO (2016). Extending the duration of hypothermia does not further improve white matter protection after ischemia in term-equivalent fetal sheep. Sci. Rep..

[68] Shankaran S (2017). Effect of depth and duration of cooling on death or disability at age 18 months among neonates with hypoxic-ischemic encephalopathy: a randomized clinical trial. JAMA.

[69] Davidson JO (2018). How long is sufficient for optimal neuroprotection with cerebral cooling after ischemia in fetal sheep?. J. Cereb. Blood Flow Metab..

[70] Davidson, J. O. et al. Limited benefit of slow rewarming after cerebral hypothermia for global cerebral ischemia in near-term fetal sheep. *J. Cereb. Blood Flow Metab*. 271678X18791631 (2018). 10.1177/0271678X18791631 PMC6827112 30092709

[71] Cho KH, Davidson JO, Dean JM, Bennet L, Gunn AJ (2020). Cooling and immunomodulation for treating hypoxic-ischemic brain injury. Pediatr. Int..

[72] Wang X (2005). Melatonin and N-acetylcysteine reduce brain injury in response to lipopolysaccharide-sensitized hypoxia-ischemia. Am. J. Obstet. Gynecol..

[73] Wang X (2007). N-acetylcysteine reduces lipopolysaccharide-sensitized hypoxic-ischemic brain injury. Ann. Neurol..

[74] Harding B, Conception K, Li Y, Zhang L (2016). Glucocorticoids protect neonatal rat brain in model of hypoxic-ischemic encephalopathy (HIE. Int. J. Mol. Sci.

[75] Savard A (2015). Neuronal self-injury mediated by IL-1beta and MMP-9 in a cerebral palsy model of severe neonatal encephalopathy induced by immune activation plus hypoxia-ischemia. J. Neuroinflamm..

[76] Yang D (2013). Intranasal delivery of cell-penetrating anti-NF-κB peptides (Tat-NBD) alleviates infection-sensitized hypoxic-ischemic brain injury. Exp. Neurol..

[77] Yang D (2013). Plasminogen activator inhibitor-1 mitigates brain injury in a rat model of infection-sensitized neonatal hypoxia-ischemia. Cereb. Cortex.

[78] Fleiss B, Nilsson MK, Blomgren K, Mallard C (2012). Neuroprotection by the histone deacetylase inhibitor trichostatin A in a model of lipopolysaccharide-sensitised neonatal hypoxic-ischaemic brain injury. J. Neuroinflamm..

[79] Xu FL (2014). Effects of erythropoietin on neuronal proliferation and apoptosis in neonatal rats after infection-induced brain injury. Zhongguo Dang Dai Er Ke Za Zhi.

[80] Traudt CM (2013). Concurrent erythropoietin and hypothermia treatment improve outcomes in a term nonhuman primate model of perinatal asphyxia. Dev. Neurosci..

[81] Wagenaar N, Nijboer CH, van Bel F (2017). Repair of neonatal brain injury: bringing stem cell-based therapy into clinical practice. Dev. Med. Child Neurol..

[82] Cho KHT (2019). Protective effects of delayed intraventricular TLR7 agonist administration on cerebral white and gray matter following asphyxia in the preterm fetal sheep. Sci. Rep..

[83] Shiow LR (2017). Reactive astrocyte COX2-PGE2 production inhibits oligodendrocyte maturation in neonatal white matter injury. Glia.

[84] Barks JDE, Liu Y, Wang L, Pai MP, Silverstein FS (2019). Repurposing azithromycin for neonatal neuroprotection. Pediatr. Res..

[85] Buller KM, Carty ML, Reinebrant HE, Wixey JA (2009). Minocycline: a neuroprotective agent for hypoxic-ischemic brain injury in the neonate?. J. Neurosci. Res..

[86] Chevin M, Guiraut C, Sébire G (2018). Effect of hypothermia on interleukin-1 receptor antagonist pharmacodynamics in inflammatory-sensitized hypoxic-ischemic encephalopathy of term newborns. J. Neuroinflamm..

[87] Herz J (2018). Interaction between hypothermia and delayed mesenchymal stem cell therapy in neonatal hypoxic-ischemic brain injury. Brain Behav. Immun..

